# Neuroimaging-pathological correlations of [^18^F]THK5351 PET in progressive supranuclear palsy

**DOI:** 10.1186/s40478-018-0556-7

**Published:** 2018-06-29

**Authors:** Aiko Ishiki, Ryuichi Harada, Hideaki Kai, Naomi Sato, Tomoko Totsune, Naoki Tomita, Shoichi Watanuki, Kotaro Hiraoka, Yoichi Ishikawa, Yoshihito Funaki, Ren Iwata, Shozo Furumoto, Manabu Tashiro, Hironobu Sasano, Tetsuyuki Kitamoto, Yukitsuka Kudo, Kazuhiko Yanai, Katsutoshi Furukawa, Nobuyuki Okamura, Hiroyuki Arai

**Affiliations:** 1 Department of Geriatrics and Gerontology, Division of Brain Science, Institute of Development, Aging and Cancer, Tohoku University, 4-1 Seiryo-machi, Aoba-ku, Sendai, Miyagi Japan; 2 Department of Pharmacology, Tohoku University School of Medicine, 2-1 Seiryo-machi, Aoba-ku, Sendai, Miyagi Japan; 3 Department of Neurological Science, Tohoku University School of Medicine, 2-1 Seiryo-machi, Aoba-ku, Sendai, Miyagi Japan; 4 Department of Pathology, Tohoku University School of Medicine, 4-1 Seiryo-machi, Aoba-ku, Sendai, Miyagi Japan; 5 Department of Nuclear Medicine and Radiology, Institute of Development, Aging and Cancer, Tohoku University, 4-1 Seiryo-machi, Aoba-ku, Sendai, Miyagi Japan; 6 Cyclotron and Radioisotope Center, Tohoku University, 6-3 Aoba, Aramaki, Aoba-ku, Sendai, Miyagi Japan; 7 Division of Community Medicine, Faculty of Medicine, Tohoku Medical and Pharmaceutical University, 1-15-1 Fukumuro, Miyagino-ku, Sendai, Miyagi Japan; 8 Division of Pharmacology, Faculty of Medicine, Tohoku Medical and Pharmaceutical University, 1-15-1 Fukumuro, Miyagino-ku, Sendai, Miyagi Japan; 9 Department of Pharmacology, Tohoku University School of Medicine 2-1, Seiryo-machi, Aoba-ku, Sendai, 9808575 Japan

**Keywords:** PSP, Monoamine oxidase, Reactive astrocyte, Tau, PET, [^18^F]THK5351

## Abstract

Recent positron emission tomography (PET) studies have demonstrated the accumulation of tau PET tracer in the affected region of progressive supranuclear palsy (PSP) cases. To confirm the binding target of radiotracer in PSP, we performed an imaging-pathology correlation study in two autopsy-confirmed PSP patients who underwent [^18^F]THK5351 PET before death. One patient with PSP Richardson syndrome showed elevated tracer retention in the globus pallidus and midbrain. In a patient with PSP-progressive nonfluent aphasia, [^18^F]THK5351 retention also was observed in the cortical areas, particularly the temporal cortex. Neuropathological examination confirmed PSP in both patients. Regional [^18^F]THK5351 standardized uptake value ratio (SUVR) in antemortem PET was significantly correlated with monoamine oxidase-B (MAO-B) level, reactive astrocytes density, and tau pathology at postmortem examination. In in vitro autoradiography, specific THK5351 binding was detected in the area of antemortem [^18^F]THK5351 retention, and binding was blocked completely by a reversible selective MAO-B inhibitor, lazabemide, in brain samples from these patients. In conclusion, [^18^F]THK5351 PET signals reflect MAO-B expressing reactive astrocytes, which may be associated with tau accumulation in PSP.

**Electronic supplementary material:**

The online version of this article (10.1186/s40478-018-0556-7) contains supplementary material, which is available to authorized users.

## Introduction

Tau positron emission tomography (PET), which provides the topographic distribution of tau aggregates in the brain, would be useful for the diagnosis of Alzheimer’s disease (AD) and for the assessment of tau burden in the clinical trials of antidementia drugs. Most studies on a tau PET radiopharmaceutical ([^18^F]AV1451) showed a robust difference between the control subjects and the patients with AD [16]. Regional distribution of [^18^F]AV1451 was correlated with tau neuropathology in *MAPT* R406W mutation carriers [37]. Tau PET also has been considered potentially useful for antemortem assessment of non-AD tauopathies, such as progressive supranuclear palsy (PSP), corticobasal degeneration (CBD), and some variants of frontotemporal lobar degeneration (FTLD) [44]. [^18^F]AV1451 PET studies have shown elevated tracer retention where tau pathology was observed frequently in patients with PSP and CBD. However, several reports have highlighted the discrepancies between antemortem PET and postmortem in vitro binding studies, particularly in non-AD tauopathies. In vitro autoradiography validation studies demonstrated that [^18^F]AV1451 failed to bind to 4-repeat tau lesions in PSP and CBD [18, 24, 38]. Recent progress in the development of second-generation tau tracers successfully reduced the off-target binding in the basal ganglia and brainstem. However, to our knowledge, no tau PET radiopharmaceutical has been fully validated against neuropathology to date [23, 43]. [^18^F]THK5351 was one of the first-generation tau PET radiotracers that was designed originally to detect tau aggregates in the form of PHF-tau in AD [11]. Clinical PET studies in PSP and CBS patients have demonstrated prominent [^18^F]THK5351 retention [2, 14, 19] in the midbrain and basal ganglia where tau pathology was observed frequently at autopsy [20, 45]. [^18^F]THK5351 binding in these areas is associated closely with disease progression because the amount of tracer retention was correlated positively with clinical severity of PSP [2]. However, recent studies have suggested the existence of off-target binding to monoamine oxidase-B (MAO-B). A single oral dose of selegiline, a selective irreversible MAO-B inhibitor, substantially reduced [^18^F]THK5351 binding in the brain of patients with PSP as well as AD [29]. In an autopsy case of AD, regional [^18^F]THK5351 binding was correlated significantly with MAO-B density as well as tau level. Therefore, [^18^F]THK5351 PET signal reflects the combination of tau pathology and reactive astrocytes in the AD brain [10]. However, what an [^18^F]THK5351 PET signal reflects in the PSP brain remains unclear.

We examined imaging-pathology correlation in two autopsy-confirmed PSP patients who showed prominent tracer retention on an antemortem [^18^F]THK5351 PET scan.

## Materials and methods

The ethics committee of the Tohoku University Graduate School of Medicine approved this study, and informed consent for neuroimaging and autopsy was obtained for each subject.

### PET and MRI scans and image analyses

PET images were acquired using an Eminence STARGATE PET scanner (Shimadzu, Kyoto, Japan). After intravenous injection of [^18^F]THK5351 (185 MBq) or [^11^C]PiB (296 MBq), dynamic PET images were obtained for 60 ([^18^F]THK5351) or 70 ([^11^C]PiB) minutes. T1-weighted magnetic resonance images (MRI) were obtained using a SIGNA 1.5-Tesla machine (General Electric, Milwaukee, WI, USA) according to a previously described method [14]. Standardized uptake value (SUV) images of [^18^F]THK5351 (40–60 min after injection) and [^11^C]PiB (50–70 min after injection) were obtained by normalizing tissue radioactivity concentration by injected dose and body weight. The regional SUV-to-cerebellar cortex SUV ratio (SUVR) was used as an index of tracer retention. SPM12 software (SPM12; Wellcome Department of Imaging Neuroscience, UCL, London, UK) was used to coregister the PET images on the MRI image. PMOD Ver. 3.7 software (PMOD Technologies GmbH, Zurich, Switzerland) was used to draw regions of interest (ROIs) on the coregistered MRI image.

### Brain tissue samples

The left hemisphere was immersed in 10% formalin for histology. The brain portions were frozen on powdered dry ice for biochemical analyses and unfixed tissue-based assays. Tissue sections of paraffin-embedded blocks were stained with Luixol fast blue and hematoxylin-eosin. Selected sections were stained with anti-tau AT8 (1:20; Innogenetics, Ghent, Belgium), anti-β-amyloid 4G8 (1:10,000; BioLegend [Signet], San Diego, CA USA), anti-α-synuclein P-syn/81A (1:100, BioLegend [Covance]), anti-TDP43 pS409/410–1 (1:5000; Cosmo Bio, Tokyo, Japan), and anti-GFAP 6F2 (1:100; Agilent [Dako], Santa Clara, CA, USA) antibodies. Brain slices 12 μm thick were generated with a cryostat (Microm HM560; Thermo Scientific, Waltham, MA, USA) using − 20 °C chamber and − 15 °C object temperatures. The sections were transferred to Matsunami Adhesive Slide (MAS)–coated glass slides (Matsunami Glass Ind., Ltd., Osaka, Japan). After drying, the sections were stored at − 80 °C. In subject 1, brain homogenates (0.1 mg/mL) additionally were prepared in phosphate-buffered saline (PBS) and stored at − 80°C until experimental use.

### Quantification of tau and glial fibrillary acidic protein (GFAP) immunoreactivity

Microscopic images from each section of paraffin-embedded blocks were captured and a threshold of optical density was obtained by Image J software (National Institutes of Health [NIH], Bethesda, MD, USA). Tau and GFAP immunoreactivity was defined as total percentage of area covered by tau and GFAP immunostaining in each ROI.

### In vitro autoradiography of [^18^F]THK5351

The brain sections were dried and were dipped in PBS for a total of 25 min and, then, preincubated in PBS containing 1% bovine serum albumin (BSA). Then, the brain sections were incubated for 30 min at room temperature with [^18^F]THK5351 (370 kBq/mL). After incubation, the sections were washed sequentially with PBS containing 1% BSA for 5 min, followed by PBS for 5 min twice. The dried sections were exposed to an imaging plate (BAS IP MS 2025 E; GE Healthcare, Little Chalfont, UK) overnight. Autoradiographic images were obtained from Typhoon FLA-9500 (GE Healthcare). To account for the binding to MAO-B in brain tissues, the reaction was incubated in the presence of MAO-B inhibitor, Lazabemide (1 μM). After post-fixation in 4% paraformaldehyde for 30 min, adjacent frozen sections were immunostained with anti-tau AT8, anti-MAO-B (1:400, Sigma-Aldrich Corp., St. Louis, MO, USA), and anti-GFAP 6F2 antibodies.

### In vitro binding assay of [^3^H]THK5351

The reaction mixture contained [^3^H]THK5351 (1 nM; specific activity, 2.96 TBq/mmol; radiochemical purity, 98.9%; Sekisui Medical, Inc., Tokyo, Japan) and brain homogenates (0.5 μg), in a final volume 200 μL. Nonspecific binding was defined in the presence of 2 μM unlabeled THK5351. The mixture was incubated at room temperature for 2 h, and separation of bound from free radioactivity was achieved by filtration under reduced pressure (Multiscreen HTS Vaccum Manifold, Multiscreen HTS 96-well 0.65 μm filtration plate; Millipore, Billerica, MA, USA), followed by three washes with PBS containing 0.1% BSA. The filters were incubated in 2 mL scintillation fluid (Emulsifier-Safe; Perkin Elmer, Boston, MA, USA), and a β counter (LS6500 liquid scintillation counter; Beckman Coulter, Brea, CA, USA) was used to count the radioactivity.

### Semiquantification of PHF-tau by immunoblotting

Immunoblotting for PHF-tau was performed according to a previously reported protocol [39]. After centrifugation (20,000 *g*, 15 min, 4 °C) of brain homogenates, the resulting pellet was dissolved in extraction buffer containing 10 mM Tris-HCl (pH 7.5), 0.8 M NaCl, 10% sucrose, 1 mM ethylene glycol-bis β-aminoethyl ether (EGTA), 2% sarkosyl, and then incubated for 30 min at 37 °C. The supernatants were collected after centrifugation at 20,000 *g* for 10 min at 25 °C. After ultracentrifugation (100,000 *g*, 20 min, 25 °C), the pellets were washed with 0.5 mL sterile saline and solubilized in sodium dodecyl sulfate (SDS)–sample buffer and, then, run on a 5–20% gradient polyacrylamide gel (SuperSep™ Ace; Wako, Osaka, Japan). Proteins were transferred to polyvinylidine fluoride (PVDF) membrane, blocked by incubation with 3% gelatin (Wako) for 10 min at 37 °C, followed by overnight incubation at room temperature with the anti-tau monoclonal antibody T46 (1:2000, Thermo Fisher Scientific), biotinylated anti-mouse secondary antibody, ABC complex (Vector Laboratories, Burlingame, CA, USA) and developed with diaminobenzidine and nickel chloride. For semiquantification of sarkosyl-insoluble tau, the three dominant bands (68, 64, and 60 kDa) were quantified by ImageJ software (Additional file 1: Figure S1). Sarkosyl-insoluble tau (PHF-tau) was expressed as ratio using cerebellum as reference.

### Quantification of MAO-B and GFAP by enzyme-linked immunosorbent assay (ELISA)

Brain MAO-B levels were quantified using a human MAO-B ELISA kit (Ab157393, Abcam, Cambridge, UK) with MAO-B standard (M7441, Sigma-Aldrich Corp.). Extraction of MAO-B was performed according to the manufacturer’s instructions. For the quantification of GFAP, brain homogenates were extracted with Tris-HCl buffer containing 0.1% Triton-X as described previously [13]. A human GFAP ELISA kit (BioVendor, Asheville, NC, USA) was used to quantify the GFAP levels.

### Statistical analysis

Spearman rank correlation coefficients were calculated to examine the association between radiotracer binding, histopathology, and biochemical data. Statistical significance was defined at *P* < 0.05. GraphPad Prism software (GraphPad, San Diego, CA, USA) was used to perform this analysis.

## Results

### Case reports

#### Subject 1

An 84-year-old right-handed male presented with memory disturbance and disorientation. One year later, standing and gait became unstable with progression of extrapyramidal signs and PSP was diagnosed clinically. PET scans were performed 2 years after the diagnosis of PSP. At the time of the PET scan, he was bedridden and the Mini-Mental State Examination (MMSE) score was 1 of 30. Neurologic examinations revealed limited vertical eye movement. The PSP rating scale score was 82. A brain MRI showed significant midbrain atrophy. A typical “hummingbird sign” was observed in the sagittal section. He died of aspiration pneumonia 295 days after the PET scan. Detailed clinical information has been described previously [14].

#### Subject 2

A 73-year-old right-handed male presented with memory disturbance. Mild cognitive impairment was diagnosed clinically 3 years after the first symptoms appeared. He gradually presented with speech impairment, stereotypical behavior, and change of food preference, and progressive nonfluent aphasia (PNFA) was diagnosed. We did not perform DNA sequencing to confirm a mutation in the *MAPT* gene. One year later, he presented with unstable gait and was prone to falls. At the PET scan, he was bedridden and the MMSE score was 1 of 30. An MRI showed diffuse brain atrophy prominent in the right anterior temporal, hippocampus, amygdala, and caudate nuclei. He died of aspiration pneumonia 79 days after PET scan.

### [^18^F]THK5351 and [^11^C]PiB PET scans

**Fig. 1:**
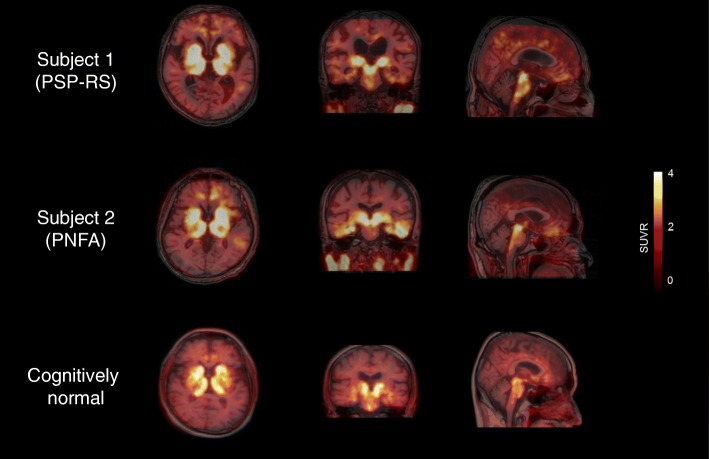
[^18^F]THK5351 PET images from two study subjects and a cognitively normal subject. The scale indicates SUVR range from 0 to 4

Figure 1 shows the [^18^F]THK5351 PET images from the two subjects. Images from a cognitively normal individual are shown for comparison at the bottom (Fig. 1). Subject 1 showed significant [^18^F]THK5351 retention in the globus pallidus and midbrain. Mild tracer retention was observed also in the other cortices, including parahippocampal and inferior temporal gyri. Subject 2 showed prominent [^18^F]THK5351 retention in the parahippocampal and inferior temporal gyri, as well as the globus pallidus and midbrain. No remarkable retention of [^11^C]PiB was observed in the neocortex in both of the subjects (data not shown).

### Neuropathological examination

Brain weight in subject 1 was 1580 g. Autopsy revealed severe atrophy in the midbrain tegmetum and pons, and subthalamic nucleus, and relatively mild atrophy in the frontal cortex, but not in the basal ganglia. Neuropathological examination revealed tau pathology in neuronal and glial cells consistent with PSP. Globose tangles were observed in the midbrain, pons, medulla, subthalamic nucleus, and nucleus basalis of Meynert. Moderate numbers of tufted astrocytes were observed also in the amygdala, motor cortex, and superior frontal gyrus. Neurofibrillary tangles were observed in the entorhinal cortex and subiculum, which corresponded to age-related Braak stage II [1]. The tau immunoreactivity density appeared greatest in the medial temporal regions, followed by the basal ganglia and frontal cortex. Small cerebral infarctions were observed in the putamen and the cerebellum. Gliosis and neuronal loss were observed also in the substantia nigra. However, amyloid-β, α-synuclein, and TDP-43 pathology were absent in this case. These characteristics were consistent with the diagnosis of definite PSP.

Brain weight in subject 2 was 920 g. Autopsy revealed severe atrophy in bilateral temporal lobes, including the hippocampus and amygdala. Brain atrophy was obviously observed in the tegmentum of the midbrain and pons. There was neuronal loss in the pigmented neurons of the substantia nigra and locus coeruleus. Brain atrophy was observed also in the globus pallidus, hypothalamic nucleus, and cerebellar dentate nucleus. AT8 immunostaining revealed abundant tau burden, including neurofibrillary tangles, globose tangles, tufted astrocytes, coiled bodies, and neuropil threads in the temporal, cingulate, frontal, striatum, globus pallidus, and subthalamic nucleus. In addition, thorn-shaped astrocytes, typical in aging-related tau astrogliopathy, were observed in the temporal lobe gray and white matter [21]. Sparse amyloid plaques were observed in the cerebral cortex (Thal phase for Aβ plaques: 2, CERAD: Sparse). Both α-synuclein and TDP-43 pathology were absent in this case. Gliosis was severe in the bilateral hippocampus and amygdala. Astrocytosis with neuronal loss was prominent in the temporal cortex, followed by the frontal and cingulate cortices. Three- and four-repeat tau-immunohistochemistry studies, respectively, revealed that these tau lesions were composed of four-repeat tau. The distribution, isoform, and morphology of tau immunoreactive lesions were consistent with atypical PSP [17], which was diagnosed as PSP-FTD.

### In vivo–in vitro correlation analyses

**Fig. 2:**
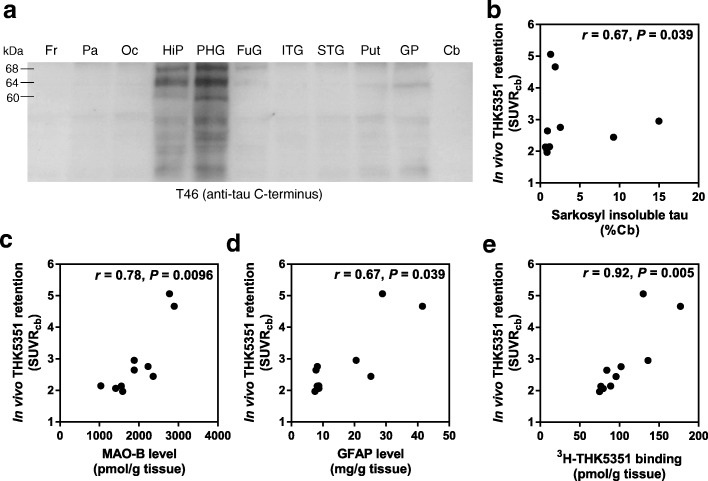
Neuroimaging, biochemical, in vitro binding correlations in subject 1 (PSP-RS). **a** Immunoblot analysis of sarkosyl-insoluble tau; 11 regions (Fr, frontal cortex; Pa, parietal cortex; Oc, occipital cortex; HiP, hippocampus; PHG, parahippocampal gyrus; FuG, fusiform gyrus; ITG, inferior temporal gyrus; STG, superior temporal gyrus; Put, putamen; GP, globus pallidus; Cb, Cerebellum) detected by T46 (anti-tau C-terminus). **b** [^18^F]THK5351 SUVR plotted against sarkosyl-insoluble tau level (%cerebellum). **c** [^18^F]THK5351 SUVR plotted against MAO-B level. **d** [^18^F]THK5351 SUVR plotted against GFAP level. **e** [^18^F]THK5351 SUVR plotted against in vitro [^3^H]THK5351 binding

**Fig. 3:**
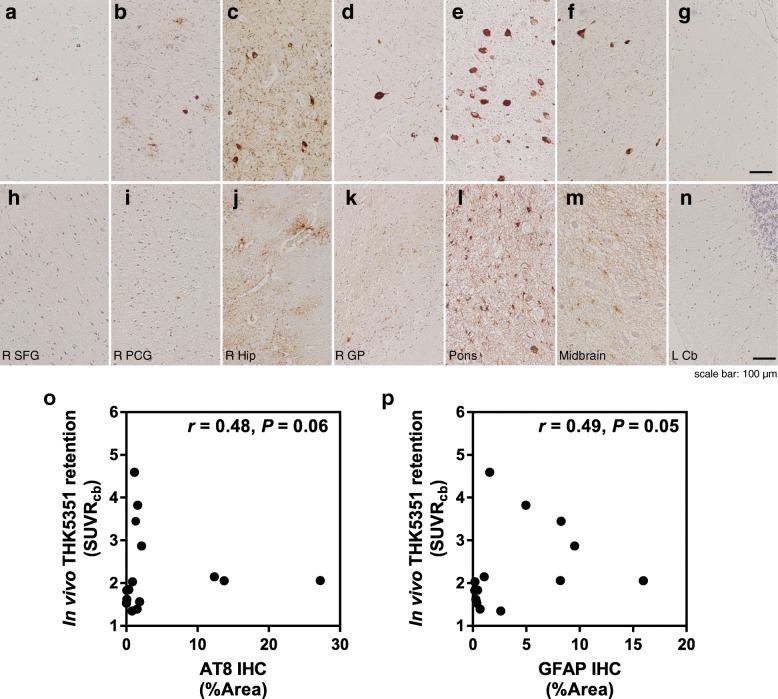
Neuroimaging-histopathologic correlations in subject 1 (PSP-RS). **a–n** Microscopic examination of tau pathology (**a–g**) and astrogliosis (**h–n**). R SFG, right superior frontal gyrus (**a, n**); R PCG, right posterior cingulate gyrus (**b, i**); R HiP, right hippocampus (**c, j**); R GP, right globus pallidus (**d, k**); Pons (**e, l**); Midbrain (**f, m**); and L Cb, left cerebellar cortex (**g, n**). [^18^F]THK5351 SUVR plotted against AT8 tau–positive areas (**o**) and GFAP-positive areas (**p**)

Biochemical analysis revealed the presence of sarkosyl-insoluble tau (68 and 64 kDa), corresponding to 4-repeat tau protein in both cases (Additional file 1: Figure S1). In subject 1, sarkosyl-insoluble tau was high in the parahippocampal gyrus and hippocampus, moderate in the globus pallidus and putamen, and low in other areas (Fig. 2a). The parahippocampal gyrus contained 3- (60 kDa band) and 4-repeat tau, suggesting that they are age-related tau. AT8 immunohistochemistry was positive in the brain sections from the same tissue (data not shown). In subject 1, in vivo [^18^F]THK5351 binding was correlated significantly with sarkosyl-insoluble tau levels determined by Western blot analysis (*r* = 0.67, *P* = 0.039; Fig. 2b). In addition, we found a strong correlation between in vivo [^18^F]THK5351 binding and MAO-B levels (*r* = 0.78, *P* = 0.0096; Fig. 2c). A significant positive correlation was observed also between in vivo [^18^F]THK5351 binding and GFAP level (*r* = 0.67, *P* = 0.039; Fig. 2d). In vitro [^3^H]THK5351 binding assay using brain homogenates also demonstrated a strong correlation between in vivo [^18^F]THK5351 retention and in vitro tracer binding in subject 1 (*r* = 0.92, *P* = 0.005; Fig. 2e). Tau and GFAP immunoreactivities in the brain sections were measured quantitatively for correlation analysis between in vivo tracer retention and histopathology (Fig. 3). We observed positive correlation trends between in vivo [^18^F]THK5351 retention and tau loads (*r* = 0.48, *P* = 0.06), and between in vivo [^18^F]THK5351 retention and GFAP immunoreactivity (*r* = 0.49, *P* = 0.05).

**Fig. 4:**
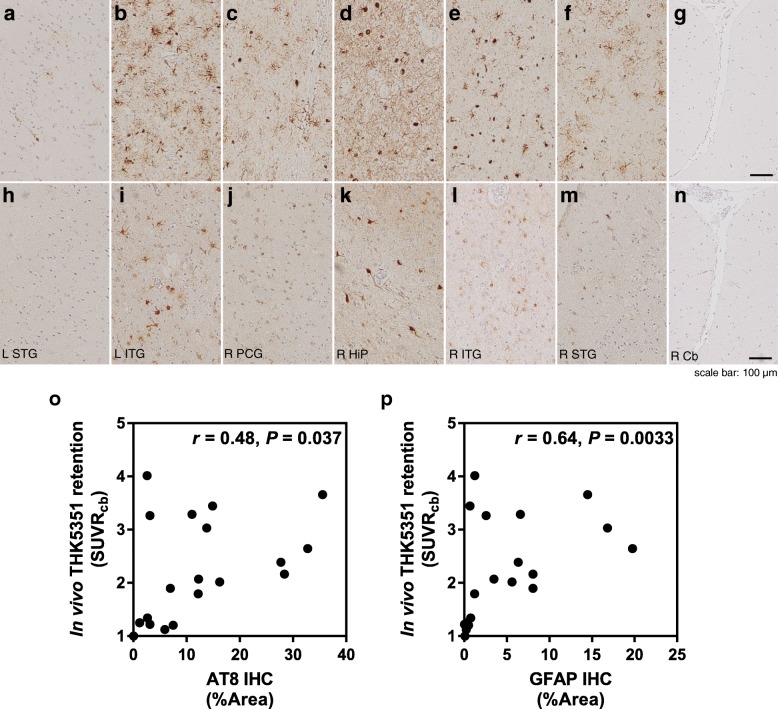
Neuroimaging-histopathologic correlations in subject 2 (PNFA). **a–n** Microscopic examination of tau pathology (**a–g**) and astrogliosis (**h–n**). L STG, left superior temporal gyrus (**a, n**); L ITG, left inferior temporal gyrus (**b, i**); R PCG, right posterior cingulate gyrus (**c, j**); R HiP, right hippocampus (**d, k**); R ITG, right inferior temporal gyrus (**e, l**); R STG, right superior temporal gyrus (**f, m**); and L Cb, left cerebellar cortex (**g, n**). [^18^F]THK5351 SUVR plotted against AT8 tau–positive areas (**o**) and GFAP-positive areas (**p**)

In subject 2, in vivo [^18^F]THK5351 binding was correlated significantly with tau-immunohistochemistry using AT8 antibody (*r* = 0.48, *P* = 0.037; Fig. 4b). Furthermore, in vivo [^18^F]THK5351 retention was correlated positively with the density of GFAP immunoreactive astrocytes (*r* = 0.64, *P* = 0.0033).

### In vitro autoradiography

**Fig. 5:**
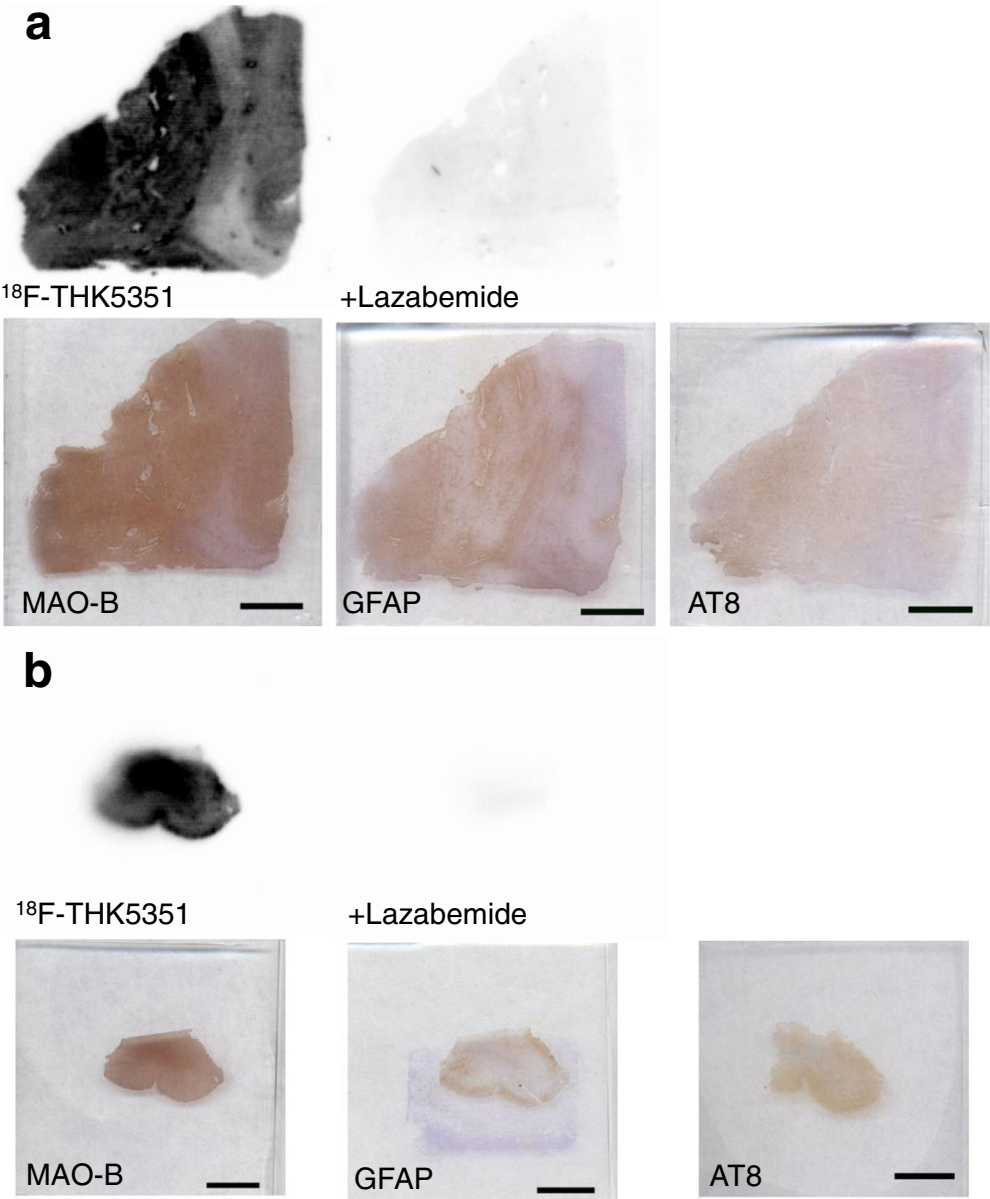
In vitro autoradiography of [^18^F]THK5351 and immunohistochemistry (MAO-B, GFAP, AT8-tau) in frozen sections from subjects. **a** Basal ganglia from subject 1 and **b** frontal cortex from subject 2. The specific binding to MAO-B was confirmed by a reversible MAO-B inhibitor, Lazabemide. *Scale bars*: 5 mm

In vitro autoradiography of [^18^F]THK5351 in frozen sections demonstrated high tracer binding in the globus pallidus as well as putamen in subject 1 and in the frontal cortex in subject 2 (Fig. 5), which was consistent with in vivo PET results (Fig. 1). These bindings were displaced completely after treatment with MAO-B inhibitor, Lazabemide [10]. The spatial pattern of [^18^F]THK5351 binding was similar to that of MAO-B immunostaining, suggesting that the target of [^18^F]THK5351 binding was MAO-B–positive astrogliosis rather than the tau aggregates in the PSP brain.

## Discussion

Tremendous efforts have been made to develop tau-selective PET radiopharmaceuticals. The first-generation tau PET tracers, such as [^11^C]PBB3, [^18^F]AV1451, and [^18^F]THK5351, have shown accumulation in the predilection site for tau deposition. Tau PET imaging recapitulated topographical regional distribution patterns similar to those reported by Braak staging of tau pathology at autopsy [44]. However, they showed nonnegligible off-target binding [4, 11, 27]. A recent human blocking study using selegiline demonstrated binding of [^18^F]THK5351 to MAO-B [29]. Furthermore, the regional [^18^F]THK5351 binding was correlated significantly with density of MAO-B in our autopsy case of AD [10]. In this study, we expanded the imaging-pathology correlation analysis to autopsy-confirmed PSP cases showing two different clinical phenotypes, Richardson syndrome (PSP-RS) and PNFA.

A patient with PSP-RS showed remarkable [^18^F]THK5351 retention in the globus pallidus and midbrain [2, 14]. The spatial distribution of [^18^F]THK5351 retention in this patient was similar to the topographical distribution of tau pathology in cases of classic PSP-RS [45]. Postmortem examination of this patient confirmed the existence of 4-repeat tau aggregates in these regions. However, imaging-pathology correlation analysis indicated a significant correlation between in vivo [^18^F]THK5351 retention and MAO-B level. Furthermore, in vitro autoradiography demonstrated that [^18^F]THK5351 binding in the globus pallidus was displaced by the MAO-B inhibitor, suggesting that [^18^F]THK5351 mainly binds to the MAO-B rather than the 4-repeat tau aggregates. In our previous study using paraffin-embedded fixed brain sections, we observed specific binding of [^18^F]THK5351 to tufted astrocytes and neurofibrillary tangles in the PSP brain [14]. However, the fixation of tissues and use of alcohol in the differentiation process may affect the tracer binding (i.e.,., diminishing the natural binding sites and/or yielding artificial binding sites) in in vitro autoradiography experiments. In this study, we performed in vitro autoradiographs of fresh-frozen sections without using alcohol and found a substantial amount of tracer binding to MAO-B. Fresh-frozen section results showed good agreement with antemortem [^18^F]THK5351 PET analysis. These results highlighted the importance of appropriate experimental procedures in the validation of PET radiopharmaceuticals.

[^18^F]AV1451 PET studies in PSP cases have shown high tracer retention in the globus pallidus and midbrain, as observed in [^18^F]THK5351 PET. However, the postmortem in vitro autoradiography did not show any significant binding of [^18^F]AV1451 in these brain regions [18, 24, 38]. The discrepancy between in vitro and in vivo PET results may be explained also by the same technical problems as those observed in THK5351. Most of these studies used high concentrations of ethanol in the differentiation process of autoradiography [5, 25, 33, 46]. High binding affinity of [^18^F]AV1451 to monoamine oxidases (MAO-A and MAO-B for *K*_d_ = 1.6 nM and 21 nM, respectively) was observed in in vitro binding assay [42]. [^18^F]AV1451 binding to MAO may be overlooked by the use of ethanol. Retrospective investigation of [^18^F]AV1451 PET in patients with Parkinson’s disease showed no significant difference between conditions before and after treatment with irreversible MAO-B inhibitors (selegiline and rasagiline) [9]. Therefore, the dominant off-target binding substrates of [^18^F]AV1451 would be MAO-A or other unknown molecules.

We observed a significant correlation between tau pathology and GFAP in both of our subjects. Tau pathology in PSP includes neurofibrillary tangles, tufted astrocytes, coiled bodies, and threads pathology [45]. A postmortem study reported that the density of GFAP correlated with that of neurofibrillary tangles, but not with tufted astrocytes in PSP, suggesting the greater contribution of neurofibrillary tangles to astrogliosis in PSP [40]. MAO-B is expressed dominantly in the mitochondrial outer membrane of astrocytes. Since elevation of MAO-B levels in the brain has been implicated in several neurodegenerative diseases, MAO-B is an attractive target as a molecular imaging marker of astrogliosis [7]. Recently, a postmortem study in parkinsonian conditions, including PSP, demonstrated that MAO-B levels elevated remarkably in the midbrain of PSP and positively correlated with astroglial markers, such as GFAP, vimentin, and Hsp27 [41], which was consistent with our observation. MAO-B PET imaging using [^11^C]L-deprenyl-D2 showed elevated tracer retention in the brain of several neurodegenerative diseases including AD [6, 15, 34]. Other investigators have reported the elevation of tracer binding in prodromal AD, but not in symptomatic AD [3, 30, 36]. However, many postmortem studies have shown the elevation of MAO-B levels in the postmortem brains of AD [8, 26, 35]. As discussed previously [41], this discrepancy might be explained by the low sensitivity of [^11^C]L-deprenyl-D2 PET. Recently, second-generation MAO-B PET tracers, such as [^11^C]SL25.1188, have been developed and showed reversible binding to MAO-B [31, 32]. Furthermore, the development of [^18^F]labeled PET tracers is ongoing [12, 28]. Our study strongly supported that [^18^F]THK5351 PET dominantly reflected the binding to MAO-B in patients with PSP. Therefore, [^18^F]THK5351 PET would be useful for in vivo assessment of astrogliosis in PSP. Future research should proceed with development of PET tracers for selective detection of astrogliosis and sensitive detection of 4-repeat tau in the human brain. -

In PSP-RS cases, ischemic changes observed in the putamen might induce reactive astrocytes. Recently, reactive astrocytes have been categorized into two different types depending on the gene expression: A1 astrocytes highly upregulated many classical complement cascades and are toxic as observed in neuroinflammation; A2 astrocytes upregulated many neurotrophic factors and are protective as observed in ischemia [22]. The GFAP showed elevated expression in both reactive astrocytes. [^18^F]THK5351 PET signal in the putamen of PSP-RS cases may reflect elevation of MAO-B levels induced by ischemia. Further studies are required to clarify MAO-B expression profiles in reactive astrocytes subtypes.

## Conclusions

Our imaging-pathology validation study demonstrated the binding of [^18^F]THK5351 to MAO-B–positive astrogliosis in the PSP brain. Therefore, [^18^F]THK5351 PET may be useful to assess astrocytosis in non-AD tauopathies.

## Additional file

Additional file 1: **Figure S1.** Immunoblot analysis of sarkosyl-insoluble tau in the study subject and an AD case detected by T46 (anti-tau C-terminus). The study subjects contained dominantly 4R tau (64- and 68-kDa tau). (PPTX 334 kb)

## Abbreviations

3R: 3 repeat
4R: 4 repeat
AD: Alzheimer’s disease
BSA: Bovine serum albumin
Cb: Cerebellum
CBD: Corticobasal degeneration
CBS: Corticobasal syndrome
ELISA: Enzyme-linked immunosorbent assay
Fr: Frontal cortex
FTLD: Frontemporal lobar degeneration
FuG: Fusiform gyrus
GFAP: Glial fibrillar acid protein
GP: Globus pallidus
HiP: Hippocampus
ITG: Inferior temporal gyrus
L Cb: Left cerebellar cortex
MAO-B: Monoamine oxidase-B
MRI: Magnetic resonance imaging
Oc: Occipital cortex
Pa: Parietal cortex
PBS: Phosphate-buffered saline
PET: Positron emission tomography
PHF: Paired helical filament
PHG: Parahippocampal gyrus
PNFA: Progressive nonfluent aphasia
PSP: Progressive supranuclear palsy
PSP-RS: Progressive supranuclear palsy Richardson syndrome
Put: Putamen
R GP: Right globus pallidus
R PCG: Right posterior cingulate gyrus
R SFG: Right superior frontal gyrus
STG: Superior temporal gyrus
SUV: Standardized uptake value
SUVR: Standardized uptake value ratio
