# Supplementary figures and images for: Neuroimaging-pathological correlations of [18F]THK5351 PET in progressive supranuclear palsy
Source: Acta Neuropathol Commun. 2018 Jun 29;6:53. doi: 10.1186/s40478-018-0556-7 (PMC6025736; doi:10.1186/s40478-018-0556-7)

## Slide 1
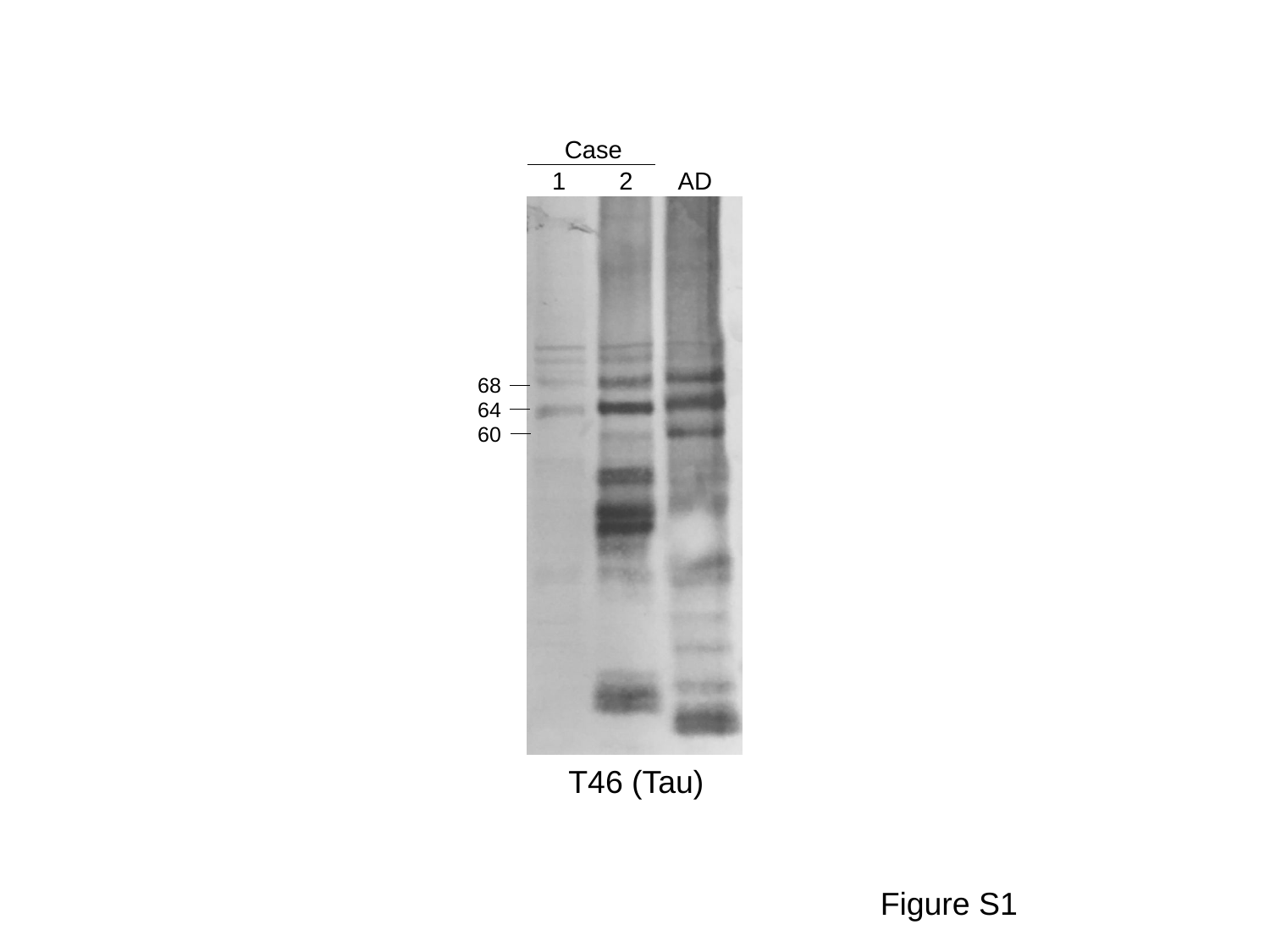

Case
1
2
AD
68
64
60
T46 (Tau)
Figure S1

Supplement: Supplementary file 1 — Figure S1. Immunoblot analysis of sarkosyl-insoluble tau in the study subject and an AD case detected by T46 (anti-tau C-terminus). The study subjects contained dominantly 4R tau (64- and 68-kDa tau). (PPTX 334 kb) [file 40478_2018_556_MOESM1_ESM.pptx]
